# Synergistic potential of natural products and exercise: unveiling molecular mechanisms and innovative therapeutic approaches for liver diseases

**DOI:** 10.3389/fnut.2025.1656048

**Published:** 2025-10-09

**Authors:** Qi Ye, Shoudu Yuan, Deliang Cai

**Affiliations:** ^1^Physical Education Institute, Chongqing College of Humanities, Science & Technology, Chongqing, China; ^2^School of Physical Education and Health, Chongqing College of International Business and Economics, Chongqing, China; ^3^Guangdong University of Foreign Studies, Guangzhou, China

**Keywords:** natural products, exercise, liver diseases, NAFLD, MASLD, hepatoprotection, metabolic health, inflammation

## Abstract

Liver diseases, particularly non-alcoholic fatty liver disease (NAFLD) and metabolic dysfunction-associated steatotic liver disease (MASLD), have grown to be significant global health issues. These conditions are strongly associated with metabolic syndrome, obesity, and diabetes. The management of chronic illnesses still necessitates lifestyle changes, such as improved diet and increased physical activity, despite advances in pharmaceutical therapies. With their numerous bioactive constituents, natural products have shown significant hepatoprotective effects through lipid metabolism, oxidative stress, and inflammatory modulation. Key natural substances, including resveratrol, curcumin, and silymarin, have demonstrated potential in clinical and experimental settings by influencing molecular pathways essential to liver health. Simultaneously, exercise interventions, specifically resistance and aerobic training, have successfully improved insulin sensitivity, decreased intrahepatic fat, and enhanced metabolic performance. Recent research suggests that the combined use of natural products and exercise represents a novel therapeutic approach. This approach may offer a therapeutic synergy that targets underlying biological mechanisms and circumvents some of the limitations of existing therapies. Furthermore, probiotic-induced microbiota modification and the gut-liver axis provide new perspectives on the holistic treatment of liver disease. This review underscores the urgent need for more studies to maximize integrative therapy approaches, identifies current research gaps, and summarizes recent developments on the combined effects of exercise and natural products in preventing and treating liver disease. These revelations open the door to creative treatments that enhance liver health and lessen the prevalence of liver illnesses worldwide, highlighting the crucial role of further research in this field.

## Introduction

Non-alcoholic fatty liver disease (NAFLD), which was recently termed metabolic-associated steatotic liver disease (MASLD) to represent its metabolic roots better, is one of the most common chronic liver diseases in the world. Due to its essential involvement in many metabolic processes, the liver is especially vulnerable to damage from lifestyle variables such as poor eating habits, sedentary lifestyles, and exposure to environmental contaminants (1). Considering the close connection between metabolic dysfunction and MASLD, creating efficient prevention and treatment plans remains a top public health goal.

Natural bioactive compounds and structured exercise programs have been shown to improve liver health and decrease the progression of MASLD (2, 3). Hepatoprotective compounds such as resveratrol, curcumin, and silymarin have shown promise in controlling oxidative stress, inflammation, and lipid metabolism (4–6). In experimental MASLD models, turmeric’s curcumin polyphenol has been demonstrated to decrease inflammation and hepatic steatosis by activating AMP-activated protein kinase (AMPK) and peroxisome proliferator-activated receptor gamma (PPARγ), two crucial regulators of energy balance and lipid metabolism (7). It has also been shown that milk thistle silymarin reduces liver enzyme levels and improves insulin sensitivity in people with metabolic syndrome and hepatic dysfunction (8). Exercise is another crucial element of enhancing metabolic health, including resistance and aerobic training. These exercise techniques enhance liver function, lower intrahepatic fat, improve insulin sensitivity, and lessen systemic inflammation—a significant contributing factor to the development of MASLD—when paired with other interventions (9–12). Exercise and naturally occurring bioactive chemicals may work in concert. Recent studies suggest that this synergy may alter the composition of the gut microbiota and the gut–liver axis, a network of communication between the liver and intestine (13).

Current pharmaceutical therapies, such as metformin and glucagon-like peptide-1 (GLP-1) receptor agonists, have demonstrated promise in improving liver histology, lowering body weight, and controlling blood sugar levels (14, 15). However, these drugs can cause gastrointestinal side effects, are costly, and have differing levels of efficacy in treating hepatic steatosis. Conversely, combining natural products and exercise offers a well-tolerated, cost-effective, and easily accessible approach simultaneously targeting multiple metabolic and inflammatory pathways (Table 1).

**Table 1:** Synergistic effects of exercise and natural products in liver diseases.

Natural products	Application model	Natural product treatment (alone)	Dose	Exercise treatment (alone)	Exercise modalities	Duration	Natural product + Exercise (Combination)	Ref.
Decaffeinated green tea	HFD-fed mice	Mild reduction in hepatic lipid accumulation and plasma ALT- Increased hepatic genes linked to cholesterol metabolism	7.7 g GTE/kg for 16 weeks	Moderate reduction in liver fat and ALT levels- Improved metabolic markers	Voluntary running wheel	16 weeks	Strongest ↓ in liver fat (↓80%) and ALT (↓92%)- Significantly increased mitochondrial biogenesis markers	(35)
*Lycium barbarum* pigment, proanthocyanins	Mice with NAFLD	TNF-α ↓, Fatty Acid Oxidation ↓, PPAR-α ↑, Protein Positivity for TNF-α↓, Protein Positivity for Fatty Acid Oxidation↓, Mitochondrial Oxidation Rate↑, Protein Positivity for PPAR-α, Antioxidant Capacity in Liver↑, Liver Lipid Accumulation↓	Not specified	TNF-α ↓, Fatty Acid Oxidation ↓, PPAR-α ↑, Protein Positivity for TNF-α↓, Protein Positivity for Fatty Acid Oxidation↓, Mitochondrial Oxidation Rate↑, Protein Positivity for PPAR-α, Antioxidant Capacity in Liver↑, Liver Lipid Accumulation↓	Aerobic treadmill exercise	7 weeks	TNF-α ↓, Fatty Acid Oxidation Regulation ↓, PPAR-α ↑, Protein Positivity for TNF-α↓, Protein Positivity for Fatty Acid Oxidation Genes↓, Protein Positivity for PPAR-α, Mitochondrial Oxidation Rate↑↑, Antioxidant Capacity in Liver↑↑, Liver Lipid Accumulation↓↓	(44)
Royal Jelly	Postmenopausal women with MASLD	Not directly assessed alone, but potential positive effects suggested	500 mg/session	Aerobic-resistance training improved PON1, HDL, reduced lipids and liver enzymes	Aerobic-resistance training (8 stations + aerobic intervals)	3 sessions/week; 35–40 min resistance + 10–15 min aerobic	Synergistic improvement in PON1, HDL ↑; oxLDL, TC, TG, LDL, ALT, AST ↓; greater liver function and lipid profile improvement compared to exercise alone	(50)
Cinnamon	Male Wistar rats fed with HFD	↓ Body weight, liver and fat mass (vs. HFD group);↓ expression of leptin gene in liver and fat tissues	200 mg/kg/day (oral gavage)	↓ Body weight, liver and fat mass (vs. HFD group);↓ expression of leptin gene in liver and fat tissues	Treadmill aerobic running, 5 days/week, progressive intensity	8 weeks	Synergistic effect: Greater ↓ in expression of leptin, body weight, liver and fat mass than either treatment alone	(54)
Cinnamon	Male rats, insulin resistant via 10% fructose for 5 weeks	↓ SREBP-1c expression (*p* = 0.045), ↑ LXRα expression (*p* = 0.024) vs. control (IRC)	200 mg/kg/day (injection)	↓ SREBP-1c (*p* = 0.032), ↑ LXRα (*p* = 0.022) vs. IRC	Aerobic treadmill training, 5 days/week, 75–80% VO₂max; 10–60 min/session	8 weeks	Strongest ↓ SREBP-1c (*p* = 0.005) and ↑ expression of LXRα (*p* = 0.0001); significantly greater ↑ LXRα vs. cinnamon (*p* = 0.044) or exercise alone (*p* = 0.048)	(53)
Chicory	Male rats with NAFLD induced by HFD (10 mL/kg)	↓ ALT, AST, ALP levels; improved hepatic histology	100 & 200 mg/kg/day (oral gavage)	↓ ALT, AST, ALP; enhanced liver tissue vs. HFD group	Treadmill walking (moderate intensity)	28 days (4 weeks)	Greatest ↓ in ALT, AST, ALP; significant hepatic tissue repair vs. HFD and single treatments	(78)
Resveratrol	Elderly male rats with NAFLD	↑ SIRT1, Lxr, Fxr expression; ↓ ALT, AST, ALP; ↓ apoptosis (*p* < 0.05)	Not specified	↓ Liver enzymes and apoptosis; modest ↑ in gene expression	Interval and continuous aerobic exercise training	Duration not specified	Synergistic ↑ in SIRT1, Lxr, Fxr expression; stronger ↓ in ALT, AST, ALP & apoptosis vs. RSV or exercise alone	(176)
Resveratrol	SAMP8 mice	↓ Apoptosis; ↑ p-Akt/Akt, Bcl2, ERK1; ↓ Bad/Cytochrome c; modulates IL-6/STAT3	20 mg/kg/daily	↓ Apoptosis, ↑ anti-aging signaling (e.g., ERK1, Bcl2); reduced liver pathology	Aerobic treadmill exercise (not precisely specified)	3 days/week for 4 weeks	Strongest ↑ in p-PI3K/PI3K, p-Akt/Akt, Bcl2; ↓ apoptosis and collagen; more effective in 6-month-old group	(94)
Resveratrol	Male Wistar rats (40–50 weeks), NAFLD model via HFD	↓ Plasma Activin A; ↑ Activin A mRNA; ↑ Follistatin mRNA; ↓ Plasma Follistatin	25 mg/kg/day (IP injection)	↓ Plasma Activin A and Follistatin; ↑ Activin A mRNA (no mention of Follistatin mRNA in exercise-alone)	Continuous and Interval (5 days/week)	8 weeks	Stronger ↑ Activin A expression and ↓ Follistatin plasma levels; ↑ Follistatin mRNA with combo groups	(95)
Resveratrol	Aged rats (20 months, 350–370 g)	↑ Bcl-2 expression; ↑ Bax expression vs. sham; ↓ Bax/Bcl-2 vs. sham	100 mg/kg/day (gavage in 1% methylcellulose)	No significant anti-apoptotic effect alone	Swimming interval training (3x/week)	6 weeks	↑ Bcl-2; ↓ Bax; ↓ Bax/Bcl-2 ratio more than all other groups—strongest anti-apoptotic effect	(96)
Resveratrol	NAFLD-induced aged Wistar rats (40–50 weeks)	↓ Serum activin A and TGF-β vs. NAFLD-only group	20 mg/kg	↓ Activin A and TGF-β levels independently	Aerobic treadmill training (continuous and intermittent)	5 sessions/week for 8 weeks	Greater ↓ in activin A and TGF-β vs. resveratrol or exercise alone	(97)
Resveratrol	Old rats with NAFLD	↓ MDA, ↓ TNF-α, ↑ Catalase, SOD, IL-10, ↓ apoptotic cells by 17.12%	25 mg/kg daily	↓ Oxidative stress and inflammation; ↑ antioxidant enzymes catalase and SOD; ↓ apoptosis (exact % not stated)	Continuous exercise; Interval exercise	3 day/week for 8 weeks	Greater ↓ in apoptotic cells (10.74% with interval, 14.85% with continuous exercise),↑ antioxidant enzymes (catalase, SOD), ↑ anti-inflammatory IL-10, ↓ TNF-α	(189)
Green tea	Male mice induced by HFD	Long-term drinking improved obesity symptoms and hepatic steatosis; inhibited NF-κB pathway and proinflammatory cytokines	Not specified	Exercise alone improved obesity symptoms and hepatic steatosis; modulated key metabolism regulators GLU2 and PPARγ	Treadmill exercise	6 days/week for 8 weeks; 6 days/week for 22 weeks	Integrated prolonged green tea + exercise synergistically prevented obesity and hepatic steatosis, ↓ SCD1, ↑ GLU2 and PPARγ, ↓ hepatic inflammation via NF-κB pathway	(36)
Yunkang 10 green tea	C57BL/6 J mice with HFD–induced metabolic syndrome	glucose↓, TC↓, TG↓, insulin↓, ALT↓, fatty liver↓, pro-inflammatory gene expression↓, Phosphorylation of IKKα/β and IκBα↓	Not specified	glucose↓, TC↓, TG↓, insulin↓, fatty liver↓, pro-inflammatory gene expression↓, Phosphorylation of IKKα/β and IκBα↓	Treadmill exercise	6 days/ week for 8 weeks	Combination showed greater improvement: ↓↓ NF-κB signaling pathway (IKKα/β, IkBα phosphorylation), ↓↓ hepatic lipid synthesis genes, ↑↑ glucose transporter gene expression in muscle	(37)
Decaffeinated Green Tea Extract (GTE)	C57BL/6 J mice fed HFD	Slight ↓ in weight gain, insulin levels, and fasting glucose; modest improvements in metabolic parameters	Not specified	Slight ↓ Body Mass, Glucose↓, Total Visceral Fat Mass, Insulin↓, IR Index↓, ↑ mt-Nd5, mt-Cytb, Ppargc1a, mt-Co3, Ppara↔, CPT1a↔, Scd1↔	Voluntary exercise	16 weeks	Significant effects: ↓ body weight (−27.1%), ↓ visceral fat (−36.6%), ↓ fasting glucose (−17%), ↓ insulin resistance (−65%),↓ plasma insulin (−65%). ↑ expression of genes involved in mitochondrial biogenesis (mt-Nd5, mt-Cytb, Ppargc1a, mt-Co3 in muscle), ↑ hepatic lipid oxidation genes (Ppara, CPT1a), ↓ lipogenesis gene (Scd1)	(190)
Tea Seed Saponins (TSS)	HFD-induced obese mice (C57BL/6 J)	↓ Body weight and adiposity; improved lipid panel (↓ TG, TC, LDL-c; ↑ HDL-c); ↓ hepatic lipogenesis gene expression	140 mg/kg·day for 4 weeks	↑ Dyslipidemia and oxidative stress markers (↑ SOD, GSH, T-AOC; ↓ ROS, MDA); slight ↑ in hepatic lipid profile	Aerobic exercise	4 weeks	Synergistic effects: ↓ Body weight and adiposity index; ↓ TG, TC, LDL-c; ↑ HDL-c; ↓ lipogenic genes (ACC, Srebp1c, Scd1); ↑ lipolysis genes (Pgc1α, Pparα, CPT1); SIRT1, ↑ p-AMPK, PGC-1α in liver; ↑ PPAR-γ & GLUT-4 in skeletal muscle; ↑ GSH, SOD, T-AOC; ↓ ROS, MDA — all contributing to reduced hepatic steatosis and oxidative stress	(114)
Garlic	Doxorubicin-induced aging rats (oxidative liver damage model)	↑ Bcl-2 (anti-apoptotic); ↓ Bax (pro-apoptotic); ↓ Bax/Bcl-2 ratio	1 ml/kg/day via gavage	↓ Bax ↑ Bcl-2 ↓ Bax/Bcl-2 ratio	Swimming training	30 min, 3 days/week for 8 weeks	Significant ↓ in apoptosis markers: ↓ Bax, ↑ Bcl-2, ↓ Bax/Bcl-2 ratio more than either treatment alone	(146)
Grape	HFD-induced obese insulin-resistant rats	↓ Insulinemia ↓ HOMA-IR ↑ Liver and muscle glycogen content	100 mg/kg daily	; ↑ Glycogen content; ↑ Insulin sensitivity in skeletal muscle; Moderate ↑ in endurence	Endurance exercise	8 weeks (3–5 times/week)	↓ Insulin resistance (synergistic) ↑ AMPK activation ↑ Muscle lipid oxidation ↑ Endurance capacity (↑ by 68% vs. EXO group) ↑ Glycogen sparing	(155)
Silymarin	HFD-induced fatty liver in rats	Slight ↓ in hepatic TNF-α (vs. HFD group); no significant reduction in hepatic leptin compared to HFD + saline	140 mg/kg body weight	↓ In hepatic leptin and TNF-α levels compared to HFD; moderate anti-inflammatory effect	Continuous aerobic treadmill running, 30 min/day, 5 days/week at 70–75% VO₂max	8 weeks	Significant ↓ in hepatic TNF-α and leptin Greater than either treatment alone. Synergistic anti-steatotic and anti-inflammatory effects from integration of aerobic exercise and silymarin supplementation	(171)
Silymarin	Dexamethasone-induced fatty liver in Wistar rats	↓ Liver enzyme levels (ALT, AST); modest ↓ in hepatocyte apoptosis	300 mg/kg per day for 8 weeks	↓ Hepatocyte apoptosis and ↑ liver histology; ↑ autophagy gene expression (AMPKα1, DRAM)	HIIT - CT	7 days DEX + intervention period	Significant synergistic effect in ↓ apoptosis and ↑ liver function and morphology; ↑ autophagy activation (↑ AMPKα1, ↑ DRAM); ↓ Caspase-9 and ↓ Mfn2 gene expression; Better histological outcomes vs. either treatment alone	(173)
Silymarin + Vitamin C	HFD-fed mouse	↑ Total antioxidant capacity, ↓ liver inflammatory biomarkers (IL-1β, TNF-α), improved histopathology	100 mg/kg	↓ liver inflammatory biomarkers, ↑ antioxidant capacity, improved histopathology	Swimming exercise	5 days/week for 8 weeks	Greater ↓ in IL-1β and TNF-α, ↑ PPARα expression, ↑ total antioxidant capacity, improved histopathology and liver enzyme regulation compared to either treatment alone	(172)

Despite promising results, few direct comparisons exist between well-established pharmaceutical treatments and natural product–exercise programs. Filling this knowledge gap is crucial to including these integrative approaches into the therapy frameworks for MASLD and other metabolic diseases. In order to inform future clinical and translational research, this review will examine underlying mechanisms, highlight current advancements in the combination of exercise and natural bioactive substances, and determine research objectives.

## Synergistic potential of natural products and exercise

The potential of combining natural products with exercise to reduce liver diseases, especially NAFLD, has drawn attention. This strategy builds on the advantages of each intervention alone and may have synergistic effects that improve liver health through various mechanisms.

## Green tea (*Camellia sinensis*)—epigallocatechin-3-gallate (EGCG)

Green tea is derived from the leaves of *Camellia sinensis*, a plant rich in polyphenolic compounds collectively known as catechins (16). The primary catechin, epigallocatechin-3-gallate (EGCG; C₂₂H₁₈O₁₁), is a potent bioactive flavonoid responsible for most green tea’s metabolic and hepatoprotective effects (17). EGCG targets insulin resistance, dysregulated lipid metabolism, oxidative stress, and inflammation (18, 19). Its primary metabolic effects result from the activation of SIRT1 and AMPK (20), which in turn enhance mitochondrial biogenesis, improve insulin sensitivity through LKB1 activation (21, 22), and decrease *de novo* lipogenesis by ACC phosphorylation and SREBP-1c/ChREBP downregulation (23). EGCG reduces pro-inflammatory cytokines (TNF-*α*, IL-6, and MCP-1), inhibits NF-κB signaling, and reduces endotoxin-induced inflammation by modulating TLR4 (17, 24). By decreasing collagen, α-SMA, and TIMP-1 expression, as well as by lowering SMAD2/3 phosphorylation and altering PI3K/Akt/FoxO1 signaling, EGCG prevents HSC activation in fibrosis (25–29). Reducing preneoplastic foci in NAFLD-related carcinogenesis, fibrosis-linked oncogenic signaling, and oxidative DNA damage can chemoprevent HCC (30–34).

Voluntary exercise and green tea extract (GTE) enhance these advantages. GTE with voluntary exercise reduced plasma ALT by 92% and hepatic TG by 56% in mice fed a high-fat diet (35, 36). They also boosted glucose and lipid metabolism through PPARα and GLUT2 activation, SCD1 suppression, and greater NF-κB inhibition (35). They also upregulated genes for fatty acid oxidation (SIRT1, peroxisome proliferator-activated receptor gamma coactivator-1 (PGC-1α), CPT1, and NRF1). Exercise demonstrated similar benefits in reducing inflammatory gene expression and steatosis when combined with Yunkang 10 green tea (YKGT) (37).

Human clinical investigations indicate the benefits of green tea for the liver and metabolism in NAFLD (38, 39). In randomized controlled trials, lipid profiles, body mass index (BMI), insulin resistance (HOMA-IR), ALT, AST, and hs-CRP were all improved by taking 500–1,100 mg/day of catechin-rich GTE capsules for 12–24 weeks (38, 39). The wealth of clinical data demonstrating green tea’s efficacy in treating non-alcoholic fatty liver disease adds confidence in the beverage’s potential as a treatment. Additional research showed that high-catechin drinks (~1,080 mg/day) decreased urine 8-isoprostane, liver-to-spleen attenuation ratio, visceral obesity, and serum ALT (40). Continuous improvements in oxidative stress markers, steatosis, and liver enzymes suggest a clinically significant benefit, even though not all metabolic parameters or body weight consistently dropped in some investigations. When combined with regular exercise, green tea catechins have been shown to target multiple stages of NAFLD, including early steatosis, inflammation, fibrosis, and even carcinogenesis.

The combined effects of voluntary exercise and decaffeinated GTE on hepatic function in rats fed a high-fat diet were investigated by Khoo et al. (35). The co-treatment demonstrated significant hepatoprotection, which led to a 92% decrease in plasma ALT and a 56% decrease in hepatic triglyceride (TG) content compared to controls. CPT1 and NRF1 were upregulated due to the combination’s mechanistic activation of SIRT1–PGC-1α signaling, which improved mitochondrial fatty acid *β*-oxidation and stimulated mitochondrial biogenesis.

Furthermore, the intervention limited fat buildup in the liver by changing hepatic metabolism away from lipid storage and toward oxidative activities. Additionally, the combined therapy increased metabolic efficiency and liver redox status, which is essential for managing diet-induced hepatic steatosis. These results show that GTE and exercise synergize lipid catabolism and mitochondrial remodeling, suggesting a viable non-pharmacological approach to NAFLD management.

Wang et al. (36) showed that in high-fat-fed mice, obesity and NAFLD phenotypes were improved after 22 weeks of exercise and green tea supplementation. In order to reduce systemic and hepatic inflammation, the intervention inhibited NF-κB signaling, which decreased the hepatic expression of pro-inflammatory cytokines such as TNF-*α*, IL-6, and MCP-1. Furthermore, the combination treatment enhanced PPARγ expression, which improved lipid partitioning and encouraged adipose tissue remodeling, and raised GLUT2, which improved hepatic glucose uptake and insulin responsiveness. Additionally, the intervention decreased hepatic *de novo* lipogenesis by downregulating stearoyl-CoA desaturase-1 (SCD1), a crucial lipogenic enzyme.

The combined effects of exercise and YKGT administration on metabolic syndrome in high-fat-fed mice, as assessed by Zhang et al. (37). They are truly remarkable. The intervention significantly reduced TG, blood glucose, total cholesterol (TC), and ALT, indicating broad hepatic and metabolic benefits. Mechanistically, the combination therapy suppressed NF-κB-mediated inflammatory signaling by downregulating the expression of hepatic pro-inflammatory genes, such as TNF-*α*, IL-6, and MCP-1. Furthermore, the intervention reduced hepatic steatosis, as evidenced by improved liver histology and reduced TG buildup. By enhancing lipid clearance and mitigating metabolic dysfunction associated with obesity, YKGT with exercise improved systemic metabolism. In the context of metabolic syndrome, these findings inspire wonder at the potential of green tea polyphenols and exercise to reduce liver inflammation and steatosis synergistically.

## Goji berry (*Lycium barbarum*)—proanthocyanidins and polysaccharides

*Lycium barbarum*, a plant in the Solanaceae family that is well known for its hepatoprotective and antioxidant properties, produces goji berries (41, 42). *Lycium barbarum* polysaccharides (LBPs) and proanthocyanidins (condensed tannins; oligomeric flavan-3-ols, C₁₅H₁₄O₆ repeating units) are mainly responsible for their bioactivity. These compounds work together to scavenge free radicals, modify oxidative stress pathways, and improve immune function (42, 43). Important for liver function, these compounds scavenge free radicals and lessen oxidative stress. Specifically focusing on NAFLD in mice, Wei et al.’s study (44) examined the combined effects of aerobic exercise and *Lycium barbarum* pigment (LBP) supplementation on the development of NAFLD in mice. The intriguing results of this study, which demonstrated significant changes in inflammatory and metabolic indicators of the liver, open up new avenues for research and potential therapeutic interventions. By increasing mitochondrial and peroxisomal fatty acid *β*-oxidation, upregulation of PPAR-*α* and its downstream targets decreased the buildup of TGs in the liver. Concurrently, NF-κB-driven inflammatory signaling was inhibited by TNF-α expression regulation, which reduced cytokine-mediated oxidative stress and hepatocellular damage.

By reducing MDA levels and increasing SOD and GSH-Px activity, the combo therapy also strengthened antioxidant defense capacity, preventing ROS-induced lipid peroxidation, which is a significant factor in the progression of NAFLD from steatosis to NASH. These results show that LBP amplifies the exercise-induced positive metabolic adaptations, such as improved lipid clearance, reduced hepatic inflammation, and strengthened redox equilibrium. Concurrently regulating PPAR-*α* and NF-κB signaling, the combination intervention offers dual defense against the lipotoxicity–inflammation–oxidative stress axis that is essential to the pathophysiology of NAFLD. These findings suggest that a combination of LBP supplementation and exercise could be a promising therapeutic strategy for NAFLD, and further research in this area could lead to the development of new treatments for this condition.

## Royal jelly (*Apis mellifera secretion*)—10-hydroxy-2-decenoic acid (10-HDA) and major royal jelly proteins (MRJPs)

Royal jelly, a nutrient-dense secretion produced by the hypopharyngeal and mandibular glands of young worker honeybees (*Apis mellifera*), is a fascinating substance (45). Its bioactive profile is dominated by 10-hydroxy-2-decenoic acid (10-HDA; C₁₀H₁₈O₃)—a unique medium-chain fatty acid with documented anti-inflammatory, antioxidant, and hormone-modulating activities (45). It also contains major royal jelly proteins (MRJPs), a family of glycoproteins with immunomodulatory and metabolic regulatory properties (45–48). These unique properties make royal jelly a subject of great interest in nutrition and hepatology (45). Vitamins, peptides (such as royalisin), and phenolic compounds (like derivatives of caffeic acid) are other ingredients that add to its medicinal flexibility (45). Through lipid peroxidation reduction, inflammatory pathway modulation, and antioxidant enzyme overexpression, royal jelly has been shown to promote liver function (48). Given that visceral fat buildup, insulin resistance, and dysregulated lipid metabolism are all exacerbated by estrogen deprivation in postmenopausal NAFLD, its hepatoprotective potential is especially pertinent (49). Because hepatic lipid homeostasis is compromised by estrogen decrease, treatments that mitigate these effects are clinically important.

Askari et al. (50) investigated the combined effects of aerobic-resistance training and royal jelly administration (500 mg per session) in postmenopausal women with non-alcoholic fatty liver disease. Over eight weeks, the combined intervention dramatically enhanced antioxidant defense, lipid metabolism, and hepatic function. A significant drop in serum ALT and AST indicated better liver integrity and less hepatic leakage. A significant rise in paraoxonase-1 (PON1) activity, an HDL-associated esterase that hydrolyzes lipid peroxides to limit oxidative stress-induced hepatocyte damage and protect the cardiovascular system, indicated improved antioxidant activity.

The study also revealed that lipid metabolism improved, with higher HDL-C and lower levels of oxidized LDL, TC, TG, and LDL-C. According to these modifications, PPARα/*γ* activity may be modulated by royal jelly constituents such as MRJPs and 10-HDA, improving fatty acid oxidation and lipid management. Reduced oxidized LDL and liver enzymes further suggest that NF-κB-mediated inflammatory signaling is being suppressed, which will lessen oxidative and metabolic stress in the liver. The combination of royal jelly and organized exercise showed better results than either regimen alone by concurrently enhancing redox balance, lipid homeostasis, and hepatocyte protection. This study underscores the potential of functional foods, such as royal jelly, to improve the metabolic adaptations of exercise. It offers a promising treatment approach tailored to the age and sex of postmenopausal women, a group at heightened risk for metabolic dysfunction, for the management of NAFLD.

## Cinnamon (*Cinnamomum verum and Cinnamomum cassia*)—cinnamaldehyde and polyphenols

Cinnamon, the dried inner bark of Cinnamomum species, particularly *C. verum* (Ceylon cinnamon) and *C. cassia* (Chinese cinnamon), holds significant promise in the field of metabolic health (51). Its pharmacological activity, primarily attributed to cinnamaldehyde (C₉H₈O), a phenylpropanoid compound, imparts cinnamon’s characteristic aroma and exerts anti-inflammatory, antioxidant, and insulin-sensitizing effects (52). Additional bioactive ingredients such as cinnamic acid, coumarin, and various polyphenols (procyanidins and catechins) contribute to its lipid-lowering and metabolic-regulating properties (53). Cinnamaldehyde, by altering metabolic processes like the activation of AMPK and the inhibition of sterol regulatory element-binding protein 1c (SREBP-1c), offers hope in improving lipid utilization and reducing hepatic steatosis.

In order to assess the effects of aerobic exercise and cinnamon extract on energy balance and hepatic lipid buildup, Dehvari et al. (54) used an high-fat diet (HFD) rat model. In line with diet-induced obesity and the advancement of NAFLD, HFD significantly increased body weight, liver and adipose tissue mass, and interfered with leptin signaling. Leptin, a hormone produced by fat cells, plays a key role in regulating energy balance and metabolism. When used alone or in conjunction with exercise, cinnamon extract—high in cinnamaldehyde and polyphenols—significantly restored leptin gene expression, indicating improved energy homeostasis and leptin sensitivity. It also decreased hepatic and adipose tissue mass, indicating decreased lipogenesis and improved lipid utilization. These outcomes demonstrate how cinnamon can combat leptin resistance and excessive fat storage, two characteristics of non-alcoholic fatty liver disease.

In mice fed high fructose, these results were validated and expanded upon by Mamashli et al. (53). Exercise and cinnamon extract decreased SREBP-1c, the master transcription factor of *de novo* lipogenesis, a process where the body converts carbohydrates into fatty acids. On their own, these treatments decreased lipogenesis. However, the combination treatment increased liver X receptor alpha (LXRα), a nuclear receptor essential for bile acid synthesis, cholesterol efflux, and lipid metabolism regulation. According to this simultaneous modulation (↓SREBP-1c, ↑LXRα), exercise and cinnamon improve hepatic metabolism by directing it away from lipid storage and toward better lipid clearance and cholesterol management.

An integrated interpretation of the research findings underscores the potential of cinnamon, particularly its phytochemicals like cinnamonaldehyde, to have pleiotropic effects. These effects include boosting lipid efflux and cholesterol metabolism via LXRα, decreasing lipogenic pathways, and modifying leptin signaling and energy balance. The activation of AMPK and PPARα by exercise, when combined with cinnamon supplementation, encourages mitochondrial biogenesis and fatty acid oxidation. These processes collectively imply that the combination of aerobic exercise and cinnamon supplementation provides a functional food–exercise synergy that can improve insulin sensitivity, reduce oxidative stress, and reduce hepatic steatosis in metabolic diseases associated with obesity and non-alcoholic fatty liver disease, instilling confidence in its therapeutic potential.

## Chicory (*Cichorium intybus L.*)—inulin and polyphenols

Chicory (*Cichorium intybus L.*) is a perennial herb of the Asteraceae family traditionally used for liver ailments for over two millennia (55–57). Its bioactivity is primarily attributed to inulin—a fructan-type polysaccharide (average formula C₆H₁₂O₆ units, *β*-(2→1) linkages) that constitutes up to 40% of chicory root dry weight (58) — and a diverse spectrum of polyphenolic compounds, including chlorogenic acid, chicoric acid, caffeic acid, and flavonoids (59–61). A prebiotic called inulin improves lipid and glucose metabolism, lowers systemic inflammation, and modifies the composition of the gut flora (62–66). Chicory polyphenols have strong antioxidant properties by scavenging ROS and upregulating antioxidant enzymes (60, 67). Numerous investigations have shown that inulin effectively increases the production of ferritin, iron-related enzymes, and genes in enterocytes (68). This improvement is linked to changes in liver enzyme levels and decreased blood ferritin levels. Chicory may help treat NAFLD, according to research that included 374 individuals with cirrhosis, hepatobiliary issues, or NAFLD (69–77). This research comprised eight studies from Iran and one from India, six of which specifically looked at various forms of chicory, including powdered seeds, water-plant extracts, chicory leaves, and powdered root extracts. The duration and dosage of the therapies varied from 4 weeks to 6 months. Most studies have shown significant changes in liver markers, suggesting that chicory may support liver health. In the study of Sefidkerdar et al. (78), the combined benefits of exercise and chicory extract on managing non-alcoholic fatty liver disease were further examined. In 56 male rats given an HFD, Sefidkerdar et al. (78) investigated the combined effects of aerobic exercise and chicory extract on NAFLD. In line with hepatocellular damage, the HFD group showed histological evidence of steatosis and increased serum liver enzymes (ALT, AST, and ALP). Chicory extract and treadmill exercise dramatically decreased liver enzyme levels, indicating better hepatocellular integrity. They also improved hepatic histology, reducing ballooning, inflammatory infiltration, and lipid droplet accumulation (78). Sesquiterpene lactones, polyphenols, and inulin-type fructans are the bioactive compounds found in chicory and are likely responsible for these protective effects. These compounds have been demonstrated to activate AMPK, which enhances fatty acid oxidation and inhibits *de novo* lipogenesis; improve gut microbiota composition and short-chain fatty acid production to lower TLR4/NF-κB-driven inflammation and endotoxemia; and provide antioxidant protection by scavenging ROS and upregulating Nrf2-dependent antioxidant enzymes (59, 60, 79–81). Exercise enhances these effects by increasing mitochondrial biogenesis and lipid oxidation and activating AMPK and PGC-1α (78). Exercise and chicory work together to enhance hepatic redox balance, insulin sensitivity, and lipid handling—all of which are essential for delaying the advancement of NAFLD (78). Although data from animals is encouraging, human validation is crucial. A clinical investigation of 374 patients with liver problems found that chicory supplementation improved liver function, further highlighting its potential (78). Based on human and animal data, chicory may have the most significant effects when combined with lifestyle changes like regular exercise. This demonstrates the potential for a nutraceutical–exercise synergy to support liver function.

## Resveratrol (3,5,4′-trihydroxy-trans-stilbene)—polyphenolic stilbene

Resveratrol (C₁₄H₁₂O₃) is a naturally occurring polyphenolic stilbene found in high concentrations in red grapes (*Vitis vinifera*), red wine, berries (e.g., blueberries, mulberries), and peanuts (82, 83). Its biological activity stems from its dual role as a potent antioxidant—scavenging reactive oxygen species (ROS) and upregulating endogenous antioxidant enzymes—and as a metabolic regulator (84, 85). This metabolic regulation is particularly significant in the context of NAFLD, as it involves the activation of AMPK, sirtuin 1 (SIRT1), and peroxisome proliferator-activated receptor-*α* (PPAR-α) signaling pathways (83, 86, 87). Animal studies have repeatedly demonstrated that resveratrol significantly reduces ALT levels, a crucial measure of liver function, which may offer protection against the liver damage associated with nonalcoholic fatty liver disease (87). The reduced LDL-C and a non-significant trend in HDL-C indicate resveratrol’s capacity to enhance lipid metabolism, which is essential for managing NAFLD and reducing cardiovascular risk (45). Additionally, resveratrol significantly lowers blood insulin and HOMA-IR values, indicating improved insulin sensitivity. This is significant because IR is crucial to NAFLD (50). Additionally, resveratrol significantly lowers blood insulin and HOMA-IR values, indicating improved insulin sensitivity. This is significant because IR is crucial to NAFLD (87). It also reduces oxidative stress and prevents oxidative damage to the liver by increasing antioxidant markers such as SOD and GSH and decreasing malondialdehyde (MDA) levels. Animal studies have also demonstrated that resveratrol significantly reduces inflammatory markers such as TNF-*α*, IL-6, and IL-1β. This is significant since the pathophysiology of NAFLD is significantly influenced by chronic inflammation (88). A preclinical study has elucidated the molecular mechanisms by which resveratrol influences NAFLD. Resveratrol has been shown to modify the AMPK/SIRT1 signaling pathway, crucial for regulating autophagy, cell death, and lipid metabolism as NAFLD worsens (89). However, it’s important to note that the current research on resveratrol and NAFLD has limitations, such as the lack of large-scale clinical trials and the need for more studies on its long-term effects. Moreover, resveratrol can counteract signals associated with inflammation by inhibiting TNF-*α*, IL-1β, and IL-6. The primary mechanism is the NF-κB signaling pathway, which is crucial for inflammation (90).

Additionally, resveratrol has been shown to enhance lipid metabolism via the PPAR-α signaling pathway (89). This pathway is essential for controlling fatty acid oxidation and overall lipid metabolism. Furthermore, resveratrol’s bioactivity on NAFLD is linked to maintaining tight gut connections, suggesting a possible role in averting gut dysbiosis, which could exacerbate liver disorders (91, 92). A more cautious viewpoint is provided by RCTs, which reveal that resveratrol did not significantly reduce ALT and AST levels in individuals with NAFLD, casting doubt on the supplement’s ability to enhance liver health in clinical settings (86). Furthermore, some research indicates a tendency for anthropometric measurements to decline. These studies, however, usually fall short of statistical significance, suggesting that resveratrol’s effects on body composition might not be strong enough to support its effectiveness. The outcomes of these trials’ lipid marker tests vary greatly, and there are no appreciable decreases in TG and TC levels. This implies that while resveratrol may influence lipid profiles, the outcomes can vary significantly (86). Furthermore, in RCTs, resveratrol did not control insulin, glucose, HOMA-IR, or TNF-*α* levels. However, it showed promise in lowering TNF-α levels, suggesting some anti-inflammatory properties that could aid in lowering inflammation linked to NAFLD (62). Preclinical research often shows the powerful effects of resveratrol, particularly in decreasing ALT and TG levels, although clinical trials have not consistently validated these findings (86). Preclinical and clinical outcomes differ significantly. These variations highlight the challenges of extrapolating results from animal studies to human contexts and the need for more study to establish the most effective dosages and durations of treatment. The dosages used in clinical trials ranged from 500 to 3,000 mg per day for 8 to 24 weeks. However, a dose/duration–effect association plot revealed that animal studies were effective at 50–200 mg/kg dosages for 4–8 weeks (86). The contradictory findings may be explained by the smaller sample sizes and varying dosages used in RCTs. In conclusion, while the evidence from RCTs is more cautious, animal studies suggest that resveratrol has hepatoprotective and lipid-regulating qualities in NAFLD. More studies are needed to identify the optimal dosages and treatment durations for resveratrol to close the gap between preclinical and clinical findings and provide more precise insights into the therapeutic potential of resveratrol for controlling NAFLD. Liver function and lipid profiles have not always improved in clinical trials. Numerous studies have shown the value of exercise and resveratrol in the treatment of NAFLD, with promising results for their combined effects. In NAFLD rats, resveratrol administration, either by itself or in conjunction with exercise, improved blood lipid profiles by reducing LDL, TC, and TG while markedly increasing HDL levels, as shown by Hajighasem et al. (93). Significantly, by downregulating the pro-apoptotic protein Bax and upregulating the anti-apoptotic protein Bcl-2, the combination treatment altered apoptotic signaling and may have a protective impact against hepatocyte death, a crucial mechanism in the development of NAFLD. Further studies confirmed these results by demonstrating that resveratrol increased the expression of hepatic SIRT1, liver X receptor (LXR), and farnesoid X receptor (FXR) both with and without exercise (70). Liver health is largely dependent on these nuclear receptors: Through AMPK and PGC-1α activation, SIRT1 improves mitochondrial biogenesis, fatty acid oxidation, and insulin sensitivity; LXR inhibits hepatic inflammation and fibrosis and increases cholesterol efflux; and FXR controls bile acid metabolism. Together, these coordinated activations reduced liver apoptosis and enhanced metabolic flexibility, bile acid balance, and lipid management. Reduced hepatocellular apoptosis, enhanced histological characteristics, and drops in serum liver enzymes (ALT, AST, and alkaline phosphatase) were evidence of restored hepatocellular integrity in conjunction with these molecular alterations (71). Research repeatedly indicates that exercise and resveratrol work in concert to enhance each other’s effects on nuclear receptor signaling, lipid metabolism, and apoptotic regulation. This dual strategy is a good option for integrative NAFLD treatment since it enhances systemic lipid balance and offers immediate hepatoprotective advantages. Senescence-accelerated mouse-prone 8 (SAMP8) mice, a model susceptible to age-related metabolic and hepatic dysfunction, were used by Suchkov et al. (94) to examine the combined effects of resveratrol and exercise. The simultaneous intervention dramatically reduced changes linked to fibrosis, such as the buildup of adipocytes and collagen in the liver. Mechanistically, the combined treatment significantly increased the activation of the PI3K/Akt pathway, a crucial signaling axis for cell survival, glucose homeostasis, and anti-apoptotic protection, while suppressing hepatic inflammation and death. The results demonstrate how exercise and resveratrol work together to support hepatocyte resilience and antifibrotic remodeling as people age and NAFLD advances. Dehghanipour et al. (73) complementary findings show that resveratrol administration and exercise training positively altered the activin A-follistatin axis in NAFLD rats. Given that follistatin counteracts the fibrogenic effects of activin A, which is linked to hepatic inflammation, fibrosis, and metabolic dysregulation, the observed rise in follistatin and fall in activin A represent a protective shift toward better inflammatory balance and hepatocellular function. All of these studies show that resveratrol and exercise together target pro-fibrotic (collagen, activin A) and pro-apoptotic pathways (Bax/Bcl-2, PI3K/Akt) in addition to modulating traditional metabolic regulators (SIRT1, AMPK, PPARs, and FXR). In NAFLD and age-related hepatopathies, this complex regulation promotes the liver’s structural integrity and functional recovery, enhancing the potential therapeutic benefits of nutraceutical–exercise synergy. According to research by Dehghanipour et al. (95), resveratrol administration plus exercise training also improved the levels of follistatin and activin A in rats with NAFLD. This implies that combining these treatments can improve liver function and inflammation. Mehboodi et al. (96) explored how resveratrol supplementation and swimming interval training affected apoptotic signaling in old rats. A crucial factor in cell survival, the Bcl-2/Bax ratio, was dramatically enhanced due to the simultaneous intervention’s strong upregulation of the anti-apoptotic gene Bcl-2 and downregulation of the pro-apoptotic gene Bax. This change suggests a potent hepatoprotective and anti-apoptotic impact, lowering age-related vulnerability to hepatocyte damage. Resveratrol plus organized exercise improved liver functional resilience in the elderly population by reducing apoptosis, indicating translational implications for halting hepatocyte loss during NAFLD and age-associated hepatic deterioration. In NAFLD, Dehghanipour et al. (97) investigated the combined effects of exercise and resveratrol administration on cytokines associated with liver inflammation and fibrosis. Central mediators of fibrogenesis and chronic hepatic inflammation, transforming growth factor-*β* (TGF-β) and activin A, were considerably decreased in the bloodstream due to the intervention. The combination approach reduced these profibrotic cytokines, reducing the pathways that support collagen deposition, extracellular matrix accumulation, and hepatic stellate cell activation—all of which are critical stages in the development of fibrosis in nonalcoholic fatty liver disease. According to the results, resveratrol and exercise work together to promote hepatoprotection by coordinating the regulation of oxidative stress, inflammation, and apoptotic signaling. This demonstrates the therapeutic potential of the combined approach for the treatment of age-related liver failure as well as NAFLD. More study is necessary to clarify the specific molecular pathways (e.g., SMAD-dependent vs. non-SMAD TGF-*β* signaling) and validate long-term efficacy in clinical populations.

## Tea seed saponins (*Camellia oleifera Abel*)—triterpenoid saponins

Tea seed saponins (TSS) are a group of triterpenoid glycosides derived from the seeds of *Camellia oleifera Abel*, a plant traditionally cultivated for tea seed oil (98). The principal bioactive molecules in TSS include camelliasaponins A, B, C, and D, each consisting of an oleanane-type triterpene aglycone linked to one or more sugar moieties (98–103). These substances can interact with membranes, bind cholesterol, and alter lipid metabolism because they are amphipathic (104). Tea seeds contain polysaccharides, polyphenols, and saponins, which have immunomodulatory and antioxidant properties (105). AMPK, a key regulator of cellular energy balance (106), activation, lipogenic transcription factor suppression (107), and intestinal cholesterol absorption inhibition (108), is one of the mechanisms by which TSS has been shown to have anti-inflammatory, antioxidant, cholesterol-lowering, and anti-carcinogenic effects (109–113). The study by Cao et al. (114) is particularly significant as it examines the combined effects of total saponins from Stauntonia chinensis supplementation and aerobic exercise in a mouse model of NAFLD induced by a HFD. The combination therapy significantly decreased body weight and adiposity, enhancing energy expenditure and fatty acid utilization, thereby boosting the body’s energy levels. Systemically, the intervention improved serum lipid profiles, increasing HDL cholesterol and decreasing TC, TG, and LDL cholesterol. At the hepatic level, TSS and AE worked in concert to attenuate steatosis by downregulating lipogenic genes, such as SREBP-1c and FAS, while upregulating genes involved in lipolysis and *β*-oxidation, promoting enhanced fatty acid catabolism. Molecular analyses showed that important metabolic regulators were activated, including p-AMPK and SIRT1, which regulate mitochondrial biogenesis and fatty acid oxidation upstream; PGC-1α, which promotes mitochondrial function and oxidative metabolism; and PPAR-*γ* and GLUT4 in skeletal muscle, which enhance insulin sensitivity and glucose uptake. This comprehensive understanding of TSS’s role in improving metabolic health enlightens us about its potential benefits in combating various health conditions.

Moreover, the significant reduction in oxidative stress provides a promising outlook on the therapeutic potential of TSS. The strengthening of antioxidant defenses, including Glutathione (GSH), superoxide dismutase (SOD), and total antioxidant capacity (TAC), along with the decrease in MDA and ROS levels, suggests a robust defense against oxidative damage and lipid peroxidation. These findings imply that TSS supplementation, in combination with aerobic exercise, holds promise in addressing oxidative stress, insulin resistance, dyslipidemia, and steatosis, key pathological features of NAFLD, by regulating AMPK/SIRT1/PGC-1α signaling and downstream metabolic and antioxidant pathways.

## Garlic (*Allium sativum L.*)—organosulfur compounds

Garlic, a member of the Liliaceae family, has been used medicinally for centuries across many cultures (115–117). Its bioactivity is primarily attributed to a diverse profile of organosulfur compounds (OSCs), including allicin, alliin, ajoene, vinyl dithiins, and S-allyl cysteine (SAC) (118–122). These compounds exhibit potent antioxidant, anti-inflammatory, hypolipidemic, anti-obesity, and insulin-sensitizing effects (122). Allicin, formed enzymatically from alliin by alliinase upon garlic crushing, is highly reactive and modulates multiple redox-sensitive pathways (122). SAC, a more stable and water-soluble compound, is recognized for its sustained antioxidant activity and NF-κB inhibition (123).

According to experimental research, garlic and its derivatives can also effectively lower hepatic steatosis, suggesting that they may have a role in liver health treatment (124). Because it may improve lipid profiles, body composition, glycemic management, and reduce inflammation and oxidative stress, garlic has garnered attention as a potential treatment for NAFLD. Numerous human studies have examined the benefits of supplementing with garlic in overweight individuals with diabetes, using varying dosages (400 mg to 400 g) and periods ranging from 26 days to 12 weeks. Most of these studies reported increases in skeletal muscle mass and changes in body composition. One study found that a high dose of 100 grams had no discernible effect on weight indices over 10 weeks, indicating response variability (125). Nevertheless, studies conducted on animals revealed that administering garlic at doses of 450, 900, and 1,350 mg/kg for 26 days caused a significant decrease in body weight, suggesting that the advantages were more pronounced in animal models (124). Additionally, garlic may enhance peripheral tissue glucose absorption, improve glycemic control, and improve the glycemic profile by reducing hepatic glucose release, most likely through PPARγ activation and improved insulin signaling (126–128). Improvements in several glycemic markers, including insulin levels and insulin resistance (IR), which are critical for managing NAFLD, have been shown in clinical studies (125, 129). Positive changes in lipid profiles have also been associated with supplementing with garlic (129). Numerous varieties of garlic, such as black garlic and garlic essential oil, have been shown to decrease TC, TG, and LDL cholesterol while increasing HDL cholesterol (127, 128, 130, 131). This lipid-modulating effect is caused by the downregulation of genes involved in lipid synthesis, including SREBP-1c, FAS, ACC, 3-hydroxy-3-methylglutaryl, and regulating the expression of CoA reductase (HMGCR). Garlic powder supplementation (400 mg/day for 12 weeks) significantly improved serum lipid profiles in individuals with NAFLD by reducing TG and LDL cholesterol while raising HDL cholesterol, according to Sangouni et al. (132). Since they have various metabolic effects, garlic’s organosulfur compounds, like S-allylcysteine and allicin, are responsible for lipid-lowering properties. Garlic increases the expression of LDL receptors, which improves the removal of circulating LDL, and inhibits HMG-CoA reductase, which lowers hepatic cholesterol synthesis. Garlic also improves fatty acid oxidation and preserves glucose–lipid balance via modulating PPAR-*α* and PPAR-*γ* activity. Hepatoprotection is further supported by its anti-inflammatory and antioxidant qualities, which include decreased lipid peroxidation and inhibition of NF-κB activation. According to these results, taking garlic supplements may help control NAFLD by enhancing lipid metabolism, lowering oxidative stress, and reducing systemic inflammation. Kim et al. (125) supplemented with fermented garlic extract and saw no discernible changes in lipid profiles. However, it should be mentioned that not all studies are in agreement. Garlic efficiently improves liver enzymes associated with non-alcoholic fatty liver disease, as evidenced by decreased ALT and AST levels (125, 128, 132–134). Garlic has anti-inflammatory and antioxidant properties. To strengthen antioxidant defenses, it lowers serum levels of pro-inflammatory cytokines (IL-6, IL-1β, and TNF-*α*) (76, 81), inhibits the expression of genes linked to inflammation, such as NF-kβ (135) and PKB/Akt, and raises levels of adiponectin, glutathione peroxidase (GPx) (128, 134) and catalase (CAT) (126, 134). Garlic has been shown in human and animal studies to decrease oxidative stress markers like MDA (126, 128, 133, 134, 136) and enhance the activity of antioxidant enzymes like TAC (125, 129, 134) and SOD (125, 127, 129, 133, 136). Since garlic appears to be a multipurpose agent that helps treat NAFLD, improve body composition, glycemic control, lipid profiles, and lower inflammation and oxidative stress, it is a beneficial dietary supplement. To guarantee the best potential health outcomes for people with non-alcoholic fatty liver disease, more research is required to standardize garlic supplementation practices and clarify the mechanisms of action. The primary categories into which garlic’s effects on NAFLD may be categorized include IR, dyslipidemia, liver enzymes and steatosis, body weight management, oxidative stress and inflammation, and gut microbiome regulation. Oxidative stress, a major contributor to NAFLD development, is exacerbated by inflammation and an overabundance of ROS. According to research, inflammation, a reduction in intracellular antioxidants, and mitochondrial dysfunction are the main causes of ROS production in the liver. Its overproduction leads to lipid peroxidation, cytokine release, and hepatocyte death, all exacerbating oxidative stress and mitochondrial dysfunction. Garlic may help reduce oxidative stress through several methods, including boosting the expression of antioxidant genes and decreasing mitochondrial dysfunction and oxidative stress agent gene expression. Organosulfur compounds (OSCs), the primary active constituents in garlic, have been shown to boost the activity of significant antioxidant enzymes like SOD, CAT, and GPx.

Additionally, SAC has been shown to decrease NF-κB activity, hence avoiding ROS-induced damage. Garlic is also necessary for treating IR, closely related to NAFLD. IR promotes hepatic steatosis and increases ROS levels by causing hyperglycemia and hyperinsulinemia due to reduced mitochondrial beta-oxidation. Garlic may aid IR by inhibiting enzymes linked to the liver’s fat synthesis and regulating lipogenesis. Additionally, garlic elevates adiponectin (134), an insulin sensitizer that enhances insulin sensitivity by activating AMPK (76) and increasing fatty acid oxidation. Garlic appears to have antihyperlipidemic effects by inhibiting intestinal microsomal TG transfer protein (MTP) expression (137) and blocking 3-hydroxy-3-methyl-glutaryl-CoA reductase (HMGR) activity (138), which is necessary for lipid production. By increasing adiponectin levels, garlic may further enhance metabolism and the lipid profile (129). Fatty acid oxidation (127, 139), lipid export (127, 139), *de novo* lipogenesis (127, 129, 139), and lipid uptake with regard to liver enzymes and hepatic steatosis are all impacted by garlic. By decreasing the activity of lipogenic enzymes (137) and altering inflammatory pathways (140–142), it raises liver enzyme levels and reduces the accumulation of hepatic fat.

Garlic contains allicin chemicals with anti-inflammatory and antioxidant properties, inhibiting NF-κB and disrupting the Jun N-terminal kinase (JNK) pathway (140, 141). Because it stimulates thermogenesis and suppresses adipogenesis, garlic has anti-obesity effects (142, 143). It may increase energy expenditure by upregulating uncoupling protein-2 (142, 143) and downregulating genes associated with fat storage and differentiation, such as PPARγ and SREBP-1c (126, 142). Moreover, garlic has a significant effect on the intestinal microbiome. Dysbiosis, which is characterized by alterations in the gut microbiota, is linked to the severity of NAFLD. Garlic’s prebiotic properties can boost the richness and diversity of microorganisms, particularly promoting beneficial bacteria like Lactobacillus (144). Despite some studies indicating that particular garlic compounds may produce fatty liver and negatively alter gut microbiota, whole garlic supplementation may improve gut microbiome variety (145). The research conducted by Shayesteh Rad et al. (146) provided additional insight into the combined benefits of swimming exercise and supplementing with garlic extract on liver function in aged rats exposed to doxorubicin-induced oxidative stress. The combined effects of swimming exercise and supplementing with garlic extract on liver function were examined by Shayesteh Rad et al. (146). In elderly rats subjected to doxorubicin-induced oxidative stress, a model that mimics age-related hepatic injury, which is significant to the evolution of nonalcoholic fatty liver disease. Forty-two male rats were split into six groups: control, doxorubicin + exercise, doxorubicin + garlic, saline, doxorubicin, and doxorubicin. For 8 weeks, the intervention involved daily administration of 1 mL/kg of garlic extract and forced swimming for 30 min, three times a week. Substantial protective benefits were seen from the combo therapy. Regarding apoptotic regulation, exercise and garlic extract improved the Bax/Bcl-2 ratio, a crucial indicator of hepatocyte survival, by raising the anti-apoptotic protein Bcl-2 and decreasing the pro-apoptotic protein Bax.

Reduction of oxidative stress was also noted, as both treatments worked in concert to improve antioxidant defenses, such as GSH, catalase, and SOD, while reducing the buildup of ROS. These modifications lessened the oxidative damage caused by doxorubicin. The combination reduced inflammation even further. In addition to reducing systemic inflammation and enhancing the hepatic microenvironment, garlic’s organosulfur compounds, including allicin, decreased pro-inflammatory cytokines and NF-κB activation. Lastly, the combination intervention provided metabolic advantages by improving lipid metabolism and raising insulin sensitivity. It decreased lipogenesis and increased fatty acid oxidation, two important processes for NAFLD management. These data show that garlic supplementation alongside regular exercise may serve as a non-pharmacological method to prevent or reduce oxidative stress–driven liver injury, particularly in older individuals predisposed to NAFLD progression.

## Grape (*Vitis vinifera L.*)—polyphenols and resveratrol

Grapes are a rich source of polyphenolic compounds, notably resveratrol, flavonoids (including quercetin, catechins, and anthocyanins), and phenolic acids, which exhibit potent antioxidant and anti-inflammatory properties (147–150). Resveratrol is particularly recognized for modulating redox balance, activating energy-sensing pathways, and regulating gene expression involved in lipid and glucose metabolism (151). Grape polyphenols have been shown to enhance insulin sensitivity, reduce oxidative damage, and protect hepatocytes from steatosis-related injury (152, 153). Exercise raises energy expenditure, encourages lipid oxidation, and improves muscle uptake of glucose. Frequent exercise has lowered the risk of liver problems and metabolic illnesses (154). A promising strategy for enhancing liver health, particularly in obesity and IR, is exercise combined with grape polyphenols. In obese rats, Lambert et al. (155) investigated the combined benefits of endurance training and grape polyphenol supplementation. Compared to either intervention alone, the study discovered that the combination therapy greatly enhanced insulin sensitivity and overall metabolic performance. An important discovery was improved energy metabolism. While fasting insulin levels and HOMA-IR scores dropped, suggesting better glucose homeostasis, the combination intervention increased the amount of glycogen stored in the liver and skeletal muscle. Another significant result was the activation of AMPK, a key regulator of lipid oxidation, energy metabolism, and endurance capacity. This activation demonstrates how the combined therapy promotes practical cellular energy usage.

By optimizing physical activity and nutrition integration, grape polyphenols and endurance exercise reduced metabolic stress linked to obesity and NAFLD and promoted more effective energy usage. These findings imply that combining structured endurance exercise with dietary supplements high in polyphenols may be a helpful non-pharmacological approach to improve liver function and lessen the consequences of metabolic disorders. As researchers, healthcare professionals, and students interested in nutrition, metabolism, and liver health, your continued engagement and contributions are crucial in furthering this research and its potential applications.

## Silymarin (*Silybum marianum L.*)—flavonolignans

Silymarin is a standardized extract from the seeds of *Silybum marianum L.* (milk thistle), containing a complex of flavonolignans (silybin A/B, isosilybin A/B, silychristin, silydianin) with potent antioxidant, anti-inflammatory, and hepatoprotective properties (156–158). It has been extensively studied for its role in NAFLD and related metabolic disorders, targeting both hepatic and systemic pathways (159–162).

Abnormalities in insulin resistance and glucose metabolism are indicated by high blood glucose levels in many NAFLD patients. HOMA-IR, blood glucose, and insulin have all been shown to decrease with silymarin (163, 164), while the exact processes underlying these benefits are yet unknown. It may reduce insulin release in response to glucose by inhibiting aldose reductase (165). Silymarin’s potent antioxidant properties, which can prevent lipid peroxidation, may help control blood sugar levels by reducing oxidative stress and improving insulin sensitivity (166). Because silymarin increases lipolysis, decreases visceral fat, and inhibits gluconeogenesis, it may help NAFLD patients with IR (167).

The significant reduction in ALT and AST levels, markers of liver damage, demonstrates silymarin’s substantial protective benefits on liver health (168). Both the advancement of NAFLD and an increased risk of acquiring conditions like cirrhosis or HCC are linked to raised ALT and AST readings. By inhibiting NF-kB and working with protein kinases to reduce COX-2, silymarin has anti-inflammatory qualities. It also protects liver cells by reducing oxidative stress, maintaining GSH levels, and regulating membrane permeability (5, 6).

Furthermore, silymarin has been shown to reduce the fatty liver index and improve the hepatic steatosis grade, which is a critical measure of the severity of NAFLD. By lowering the expression of genes involved in *de novo* lipogenesis, such as SREBP-1c, FAS, and ACC1, and by guarding against oxidative stress, inflammation, and steatosis—all of which are critical elements in the development of the disease—sililymarin improves lipid metabolism in NAFLD (169, 170).

However, drug combinations may affect silymarin’s effectiveness, highlighting the need for cautious NAFLD treatment. The findings suggest that silymarin therapy should be started early to decrease the disease’s progression, particularly in patients with NAFLD rather than NASH. This potential of silymarin to prevent disease progression should inspire hope and optimism about its therapeutic benefits. In Wistar rats with fatty liver disease, Shahamat et al. (171) examined the combined benefits of aerobic exercise and silymarin administration. According to the study, this combined intervention significantly reduced hepatic inflammation, as shown by lower levels of TNF-*α* and leptin. These modifications show that the inflammatory pathways linked to liver injury are effectively suppressed.

Along with lowering inflammation, aerobic exercise improved metabolism, which enhanced the hepatoprotective advantages of silymarin. Exercise enhances hepatic health by promoting fat oxidation, reducing systemic inflammation, and improving insulin sensitivity. These results collectively imply that silymarin supplementation with aerobic exercise may provide a synergistic strategy for preventing liver damage in NAFLD by modifying metabolic and inflammatory pathways. The combined effects of silymarin and exercise, greater than the sum of their individual effects, demonstrate the power of combined therapies in enhancing the therapeutic benefits of silymarin in NAFLD management.

Among its many metabolic benefits, aerobic exercise enhances insulin sensitivity, fat oxidation, and inflammation reduction. The hepatoprotective effects of silymarin in conjunction with exercise were investigated by Aghaei et al. (172) and Shahamat et al. (171). Both studies showed that the combined therapy had a synergistic impact, significantly reducing hepatic inflammatory markers such as leptin, TNF-*α*, and IL-1β, more than either intervention alone. This impressive result underscores the power of interventions working together to alleviate liver inflammation.

Exercise and silymarin increased antioxidant capacity while also lowering inflammation. This boost in antioxidant defenses aids in preventing liver damage brought on by oxidative stress, which is a significant contributing cause to the development of NAFLD. The combined intervention promotes cellular health and overall liver function by lowering oxidative damage. Lipid metabolism was also enhanced by silymarin and exercise. According to the findings, PPAR-*α* expression, a crucial regulator of fatty acid oxidation, was upregulated, which helped to minimize the accumulation of fat in the liver. According to these results, the dual strategy encourages the liver to handle lipids more healthily while lowering oxidative stress and inflammation. The varied exercise regimens showed that various aerobic exercises can enhance silymarin’s benefits. Aghaei et al. (172) used swimming exercise, while Shahamat et al. (171) used treadmill jogging for 30 min at 70–75% VO₂max. Notwithstanding these variations, silymarin supplementation and both types of aerobic exercise had complementary advantages.

Additionally, a more thorough improvement in liver function was noted when silymarin and exercise were combined with other nutrients like vitamin C (172). This combination may provide additional antioxidant support, further strengthening the liver’s defenses against oxidative stress. This comprehensive strategy, combining silymarin with exercise and other nutrients, should reassure the audience about the effectiveness of the treatment in controlling NAFLD, especially in high-risk groups like the elderly or those following high-fat diets. To summarize, this comprehensive strategy provides a powerful tool to lower inflammation, strengthen antioxidant defenses, enhance lipid metabolism, and safeguard liver health.

## Mechanistic overview

A complex but intricate web of biochemical pathways supports the synergistic effects of exercise and natural products in reducing the severity of liver diseases like NAFLD/MASLD. These pathways regulate inflammation, oxidative stress, and energy metabolism, with AMPK, SIRT1, PPAR (*α*/*γ*), and NF-κB as the central signaling nodes. Their actions combine to create additive or even supra-additive effects and hold significant implications for potential interventions in liver disease reduction.

## AMPK activation—the energy master switch

AMPK operates as a crucial metabolic ‘master switch,’ sensing the energy level of cells and activating when the AMP/ATP ratio rises in situations of increased energy demands, such as during exercise, or when bioactive substances like resveratrol, silymarin, and tea seed saponins directly stimulate it (114, 173). Its activation removes the inhibition of CPT1, thereby increasing mitochondrial fatty acid import, as it phosphorylates and deactivates ACC, reducing malonyl-CoA levels (114). AMPK’s role in lipid metabolism is evident in its inhibition of lipogenesis, achieved by downregulating the production of FASN and SREBP-1c (174). Additionally, it enhances insulin sensitivity by promoting glucose absorption in muscle tissue through the upregulation of glucose transporter type 4 (GLUT4). However, the most intriguing aspect of AMPK’s function is its role in promoting mitochondrial biogenesis. By activating the PGC-1α, AMPK demonstrates its potential in boosting the body’s oxidative phosphorylation capability (175).

## SIRT1 modulation—linking energy status to gene expression

SIRT1, a NAD⁺-dependent deacetylase, is activated by polyphenol intake (e.g., resveratrol and green tea catechins) and endurance or interval training, primarily through an increased NAD⁺/NADH ratio (114, 176). Once activated, SIRT1 enhances mitochondrial function by deacetylating and activating PGC-1*α*, which collaborates with AMPK to drive mitochondrial oxidative metabolism (114). SIRT1 also plays a pivotal role in reducing inflammation by deacetylating the p65 subunit of NF-κB, thereby reducing the transcription of pro-inflammatory genes, offering a potential solution for inflammatory conditions (177). SIRT1 also contributes to metabolic reprogramming by indirectly modulating PPAR-α and PPAR-*γ* target gene expression, thereby optimizing lipid handling and glucose metabolism. Most notably, it promotes longevity and stress resistance by activating FOXO transcription factors, which in turn increase catalase and SOD activity, thereby strengthening antioxidant defenses (178–180).

## PPAR signaling—lipid oxidation and storage regulation

Signals from nutrients are converted into coordinated lipid metabolism programs by ligand-activated transcription factors called peroxisome proliferator–activated receptors (PPARs) (181, 182). Hepatocytes and skeletal muscle benefit from PPAR-*α*’s promotion of *β*-oxidation, which lowers hepatic lipid buildup (182). Its activation can be triggered by fatty acid derivatives, lycopene-rich wolfberry pigment, and tea seed saponins, either directly or indirectly via AMPK and SIRT1 signaling (183). The comprehensive nature of lipid metabolism regulation is underscored by the role of PPAR-*γ* in controlling adipocyte development, lipid storage, and insulin sensitivity, which makes the redistribution of lipid stores away from the liver easier. Together with PPAR-activating natural compounds, endurance training has a synergistic impact that speeds up hepatic lipid clearance by independently activating PPAR-*α* in the muscle and liver.

## NF-κB inhibition—breaking the cycle of inflammation

Hepatic damage is sustained by nuclear factor kappa-light-chain-enhancer of activated B cells (NF-κB), a crucial transcription factor that promotes the development of adhesion molecules and pro-inflammatory cytokines, including TNF-α and IL-6 (36). Bioactive substances such as green tea catechins, silymarin, garlic ingredients, and certain flavonoids can directly limit their action by blocking NF-κB nuclear translocation, inhibiting upstream kinases (IKKα/*β*), and preventing IκBα phosphorylation (5, 6, 36, 37). However, the role of SIRT1 in deacetylating the NF-κB p65 subunit further strengthens this anti-inflammatory impact (184). It provides a long-lasting inhibition of inflammatory signaling, offering a sense of reassurance about the potential effectiveness of these interventions. The intriguing potential of aerobic and resistance exercise to indirectly affect NF-κB activity by reducing oxidative stress and activating AMPK, which helps lower hepatic inflammation in concert, highlights the importance of lifestyle interventions.

## Integrated mechanistic model—a converging network

**Figure 1:**
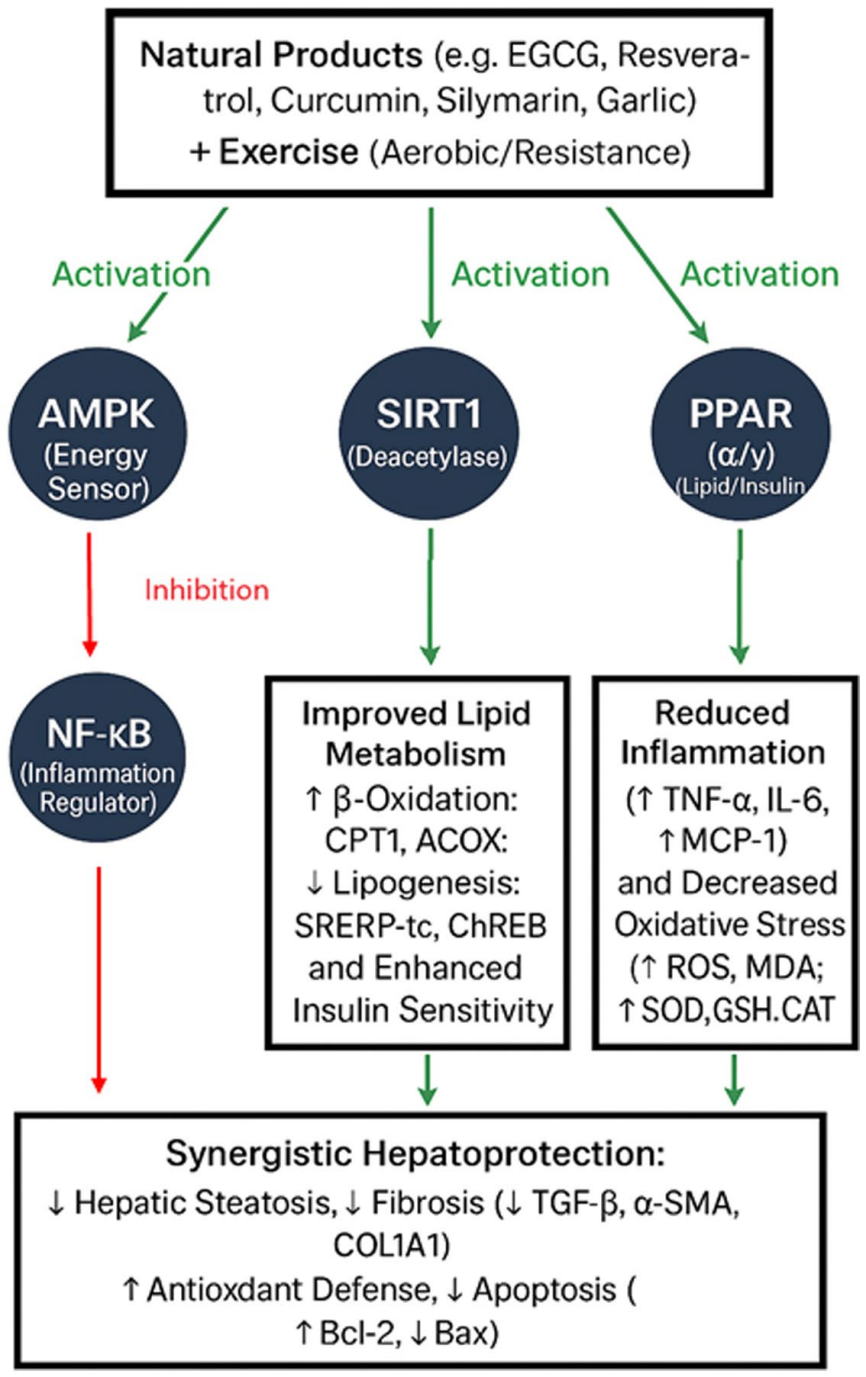
Integrated mechanistic model illustrating how key signaling pathways—AMPK, SIRT1, PPAR (α/γ), and NF-κB—interact to mediate the synergistic hepatoprotective effects of natural products and exercise in NAFLD/MASLD. Co-activation of AMPK and SIRT1 enhances mitochondrial biogenesis, fatty acid *β*-oxidation, and antioxidant defenses, while concurrent PPAR activation promotes lipid clearance and NF-κB inhibition suppresses hepatic inflammation. This converging network results in coordinated metabolic reprogramming and inflammation resolution, producing additive or supra-additive therapeutic benefits.

Co-activation of AMPK and SIRT1 is the biochemical foundation of the synergy between exercise and natural products, promoting stronger antioxidant defenses, quicker *β*-oxidation, and increased mitochondrial biogenesis (Figure 1) (114). A reciprocal interaction supports this upstream convergence, whereby SIRT1 deacetylates LKB1, a kinase that activates AMPK, and AMPK increases NAD⁺ availability to promote SIRT1 activity (114). These signals work in concert to cause a metabolic change in which hepatocytes and muscle cells are guided toward oxidative metabolism by the enlightening role of PPAR activation, which lowers ectopic lipid accumulation. At the same time, NF-κB regulation eliminates inflammation and breaks the cycle of lipotoxicity-induced damage. Both direct antioxidant actions and SIRT1-mediated deacetylation accomplish this. Synergistic hepatoprotection results from a multi-axis reprogramming of inflammatory state and hepatic metabolism (Figure 1). According to human and animal research evidence, exercise combined with natural products reliably reduces hepatic steatosis, serum transaminases, and inflammatory markers more than either intervention alone (Table 1).

## Enhanced apoptotic regulation

The controlled process of cell death, known as apoptosis, helps maintain the equilibrium of cellular processes. It is required to eliminate damaged or superfluous cells (185). The development of NAFLD is thought to be influenced by apoptosis, a mechanism of programmed cell death (186). In NAFLD, apoptosis can be triggered by oxidative stress, inflammation, and IR. In order to lessen NAFLD and its related problems, apoptosis management is advantageous (186). It has been shown that exercise and natural supplements improve the balance between pro-apoptotic and anti-apoptotic proteins, increasing liver cell survival. This method can lessen the apoptosis in liver cells, which is frequently increased in NAFLD. The possible impact of resveratrol on apoptosis in the setting of non-alcoholic fatty liver disease has been investigated (87). Since oxidative stress and inflammation are key factors in the development of NAFLD, resveratrol’s antioxidant qualities can help lower them (187).

The activation of pro-apoptotic pathways may be suppressed by resveratrol via reducing inflammation and oxidative damage. When resveratrol and exercise were combined, Hajighasem et al. (93) found that the combination increased Bcl-2 levels and decreased Bax levels. In NAFLD, this alteration in the Bax/Bcl-2 ratio—which signifies a move toward an anti-apoptotic effect—is crucial for reducing liver damage. This observation is important in the context of apoptosis modulation in NAFLD because a lower Bax/Bcl-2 ratio indicates a more protective environment in the liver and a lower risk of apoptosis. The association between apoptosis and the combined benefits of swimming and resveratrol on liver function in aged rats was examined in the study by Mehboodi et al. (96). Aging negatively impacts liver health, which is associated with a loss of physiological function, physical activity, and diet. Since the liver typically suffers from increased cell turnover and damage as people age, apoptosis is significant. In this experiment, 32 rats weighing between 350 and 370 grams and about 20 months of age were divided into four groups: a sham group, a training group, a resveratrol group, and a combination training and resveratrol group. For 6 weeks, the resveratrol groups were given a daily dose of 100 mg/kg of resveratrol, which was gavaged after being dissolved in 1% methylcellulose. In comparison, the training groups spent the same time swimming three times a week. Significant alterations in apoptotic markers were found, and both the resveratrol and combined training plus resveratrol groups had significantly greater levels of expression of the anti-apoptotic gene Bcl-2 than the sham and exercise-only groups. In contrast, the combination therapy group had lower levels of the pro-apoptotic gene Bax than the resveratrol-only group. In contrast to the sham group, it was higher in the resveratrol group at the same period. According to the study, a lower ratio in the combined therapy group indicated a more protective environment in the liver and a lower risk of apoptosis, highlighting the Bax/Bcl-2 ratio as a crucial predictor of the apoptotic process. The encouraging findings of this study raise the possibility of better liver function by indicating that resveratrol and exercise together may strengthen the liver’s defenses against cell death.

A natural compound from the milk thistle plant called silymarin has been investigated for its possible effects on NAFLD-related apoptosis. Silymarin may improve liver function and reduce fibrosis in people with nonalcoholic fatty liver disease, according to research. The balance between pro-apoptotic and anti-apoptotic proteins is impacted by its regulation of several apoptotic pathways. While potentially inducing apoptosis in injured liver cells, this activity encourages the survival of healthy liver cells. Exercise and silymarin decrease apoptosis-related indicators, including Mitofusin 2 (Mfn2) and caspase-9. Important outcomes from this combination were emphasized by Andani et al. (173), who reported a considerable drop in caspase-9 levels, indicating a decreased activation of the apoptotic pathway and enhanced cell survival. Because Mfn2 is necessary for mitochondrial function, its decrease with caspase-9 suggests that mitochondrial health may be improved. These encouraging results suggest that silymarin plus exercise may work in concert to improve diseases like NAFLD, where apoptosis is a key factor in the development of the disease. This combination may provide a treatment strategy to reduce liver damage and encourage cell survival, giving hope that lifestyle modifications may help manage liver-related health conditions.

Due to its anti-inflammatory and antioxidant qualities, garlic can help lower oxidative stress in the liver, which is a significant factor in developing NAFLD. According to some research, garlic supplements may help enhance liver function and lessen hepatic cholesterol buildup, two important aspects of managing nonalcoholic fatty liver disease. Swimming exercise raised Bcl-2 and decreased Bax levels in the study by Shayesteh Rad et al. (146), suggesting a lower risk of apoptosis and better liver health.

Likewise, supplementing with garlic resulted in higher Bcl-2 and lower Bax levels, indicating that garlic may help prevent liver cells from dying, especially under oxidative stress. Supplementing with garlic extract and swimming together increased the positive effects on the Bax/Bcl-2 ratio and considerably decreased liver damage brought on by doxorubicin-induced oxidative stress. Both therapies support the balance between pro-apoptotic and anti-apoptotic signals by reducing oxidative stress, which enhances cell survival and function. This demonstrates how garlic may have the ability to shield liver cells from apoptosis, offering comfort and defense against NAFLD.

## Promotion of autophagy

Lipid buildup in the liver is a hallmark of NAFLD. Ectopic lipid accumulation is a defining feature of hepatic steatosis that is frequently linked to non-alcoholic fatty liver disease. Autophagy plays a key role in the metabolism of lipid droplets, which are mainly made up of TG and cholesterol esters in the liver. Impaired hepatocyte autophagy of these lipid droplets plays a potential pathophysiological role in NAFLD. Nonetheless, there is hope for the future of NAFLD treatment due to the possibility of encouraging autophagy as a successful tactic to reduce hepatic lipid buildup. Several natural compounds are thought to help lessen this accumulation by triggering autophagy, at least in part. Furthermore, some research shows that combined treatments can increase the expression of autophagy-related proteins, including damage-regulated autophagy modulator (DRAM) and AMPKα1, which help to remove damaged liver cells and encourage liver regeneration.

The natural substance silymarin is known to have liver-protective properties. According to the study by Andani et al. (173), silymarin used in conjunction with exercise training greatly enhanced liver protection. The study comprised 49 male Wistar rats that were split up into groups, such as a control group, a group with DEX (dexamethasone-induced fatty liver), and groups that were given silymarin supplements, continuous training (CT), or high-intensity interval training (HIIT). Autophagy-related genes, such as AMPKα1 and DRAM, were examined for expression using real-time PCR. Both CT and HIIT reduced liver damage and produced favorable changes in liver structure.

Combining silymarin with exercise led to greater liver protection and elevated autophagy-related proteins AMPKα1 and DRAM levels. This gives us hope that silymarin may be used to treat NAFLD. An essential energy sensor that controls energy balance in cells is AMPK. When triggered, it promotes catabolic mechanisms, such as autophagy, to provide resources and energy during stress or nutrient shortage. It is implied by the increase in AMPKα1 expression that silymarin and exercise together improve cellular energy sensing and promote autophagic activities, which help eliminate damaged proteins and organelles. In order to generate autophagosomes, which are necessary for breaking down cellular components, DRAM is a protein needed for the autophagy process. Increased DRAM levels imply that this combination may improve autophagic flux, cell survival, and general health by aiding the breakdown of damaged cellular components.

## Clinical implications

To manage NAFLD and associated metabolic diseases, this section aims to offer practical, evidence-based recommendations that are easily incorporated into therapeutic practice. The suggested dosages are not merely theoretical; they are based on results from preclinical animal research and clinical trials, with modifications made to guarantee their suitability for use in human beings. For example, it has been demonstrated that GTE, which is high in catechins, can enhance lipid profiles, insulin resistance markers, and liver enzyme levels (ALT, AST). The recommended doses of 500–1,100 mg daily for 12–24 weeks are advised based on the optimal balance between efficacy and safety. Similarly, royal jelly has shown positive benefits on lipid metabolism and liver function in NAFLD patients when given 500–1,000 mg before exercise sessions, with the dosage chosen to align with the body’s metabolic response to exercise (50). There is also discussion of other natural compounds, including garlic (129, 132), tea seed saponins (114), resveratrol (86, 176), cinnamon extract (53, 54), chicory (69, 78), grape polyphenols (155), and silymarin (171–173), along with recommended dosage guidelines based on the existing scientific data. Clinicians are urged to use standardized extracts to guarantee constant amounts of active ingredients, and it is underlined that dosages should be customized based on patient weight, individual tolerance, and clinical response.

Evidence indicates that the best time to take supplements is right before exercise, ideally 30 to 60 min beforehand, as this aligns peak bioavailability with exercise-induced metabolic and anti-inflammatory pathways, including SIRT1 and AMPK activation, and maximizes their synergistic effects (114, 155). This not only maximizes the physiological advantages, boosting insulin sensitivity, decreasing inflammation, and increasing fat metabolism, but also empowers the patients to participate in their treatment actively. Exercise regimens that support these interventions include swimming, resistance training with aerobic intervals, HIIT, and aerobic training at a moderate intensity (70–80% VO2max) three to five times a week for 30 to 60 min (10–12). The role of exercise in the treatment plan is crucial, and numerous studies show that the best results are obtained after 12–24 weeks, and clinical data support the need to continue these combined interventions consistently for at least 8 weeks (114). With such a consistent strategy, liver function, metabolic health, and overall patient outcomes can improve. With the potential to significantly improve the management and prognosis of liver illnesses in clinical settings, this extended, integrated framework gives medical professionals a useful, evidence-based road map for synergistically combining natural products and exercise.

## Limitation

The study identifies several significant research gaps that impact the clinical use of natural products and exercise treatments for liver disorders. It points out that many human trials have used very small sample sizes, usually between 17 and 80 people, which restricts the findings’ statistical power and potential to be applied to larger groups. The study emphasizes the need for larger, multicenter RCTs to verify efficacy in various groups. Furthermore, the study highlights that most trials have had extremely short follow-up periods (usually 8–24 weeks), which has hampered our understanding of the therapies’ long-term safety and sustained efficacy. To assess the durability of benefits and potential hazards, particularly in halting the progression of more serious liver disorders such as HCC or NASH, longer-term, longitudinal trials lasting more than a year are necessary. The study also reinforces the significant issue of the absence of thorough long-term safety data, especially about high dosages of silymarin and GTE (over 1,000 mg/day), which have been sporadically linked to uncommon instances of hepatotoxicity. Therefore, the study emphasizes the importance of standardized extracts and ongoing patient monitoring to reduce adverse effects. The study also points out that some preclinical benefits, such as lower ALT and TGs, have not been consistently seen in clinical trials, and many animal studies have used doses that are hard to replicate in people, creating translational hurdles. Future research, the study suggests, must concentrate on human-equivalent doses and standardized formulations because variations in human metabolism, bioavailability, and illness models complicate direct translation from animal findings. Lastly, the study highlights the challenge of directly comparing results or determining the best intervention combinations due to the heterogeneity in study protocols, which includes variations in natural product formulations (e.g., isolated GTE versus whole garlic preparations) and variations in exercise types (aerobic, resistance, and HIIT). The study concludes that standardized, carefully thought-out protocols are required for clinical practice to progress and fill these gaps.

## Future directions

A promising strategy for treating liver ailments, especially NAFLD and associated metabolic abnormalities, is the combination of natural products and physical activity. Future research should concentrate on a few crucial areas to improve the effectiveness and application of these therapies. First, to ascertain the generalizability of results and pinpoint particular demographic responses to therapies, larger clinical studies with various populations—including different age groups, ethnic origins, and concomitant conditions—are crucial (73, 188).

Further investigation into the molecular processes that underlie the complementary benefits of exercise and natural products is also essential, with a particular emphasis on pathways like AMPK, SIRT1, and PPAR signaling that are essential for controlling inflammation and lipid metabolism (145, 153). If these pathways are understood, it will be easier to optimize dosages and combinations for optimum therapeutic impact. Furthermore, creating individualized treatment plans based on lifestyle, metabolic, and genetic variables may improve the efficacy of natural supplements and exercise. Personalizing interventions to each patient’s unique profile, including genetic susceptibilities to liver diseases, may also improve results and adherence (95, 97).

To investigate the sustainability of benefits and the possibility of slowing the progression of the disease to more serious conditions like cirrhosis or HCC, longitudinal studies evaluating the long-term effects of dietary and exercise interventions on liver health and metabolic parameters are also required (129). Research on the relationship between gut microbiota, nutrition, and liver health has also been more popular recently; future studies should look into how particular dietary patterns and natural products affect the composition of the gut microbiota and how that affects liver health. This might result in novel probiotic or prebiotic approaches that support natural product and exercise therapies (92). Finally, as knowledge of possible synergistic effects among different substances may result in more robust therapy alternatives and better management of liver illnesses, more research should examine the effects of mixing diverse natural products with exercise (155). Future studies tackling these topics can significantly advance our knowledge of and ability to treat liver illnesses using comprehensive strategies incorporating natural remedies and lifestyle changes.

## Conclusion

A novel and promising strategy for the prevention and treatment of fatty liver diseases (FLDs), such as NAFLD and alcohol-related liver disease (ALD), is the synergistic use of natural products with exercise. Polyphenols, flavonoids, and omega-3 fatty acids are natural compounds that target important pathways in the pathophysiology of FLD, such as lowering oxidative stress, regulating inflammation, boosting mitochondrial function, and optimizing lipid metabolism. By controlling energy balance, improving insulin sensitivity, and lowering hepatic fat storage, exercise enhances these effects and offers a variety of other advantages. Through common molecular pathways like PPAR signaling, AMPK activation, and gut-liver axis regulation, the combination of these therapies enhances their individual therapeutic effects. These processes support better metabolic health, less fibrosis, and enhanced liver function. This synergistic strategy is supported by preclinical and developing clinical evidence. However, it also emphasizes the need for more study to refine intervention procedures, such as choosing certain natural substances, efficient dosages, and individualized exercise plans based on patient needs. This appeal for more study highlights the current work in this area and involves the audience in the scientific method. Healthcare providers can provide patient-centered, sustainable solutions to lessen the worldwide burden of FLDs by including natural products and organized exercise into comprehensive management plans. By improving long-term health outcomes and lowering the likelihood of disease progression, this innovative therapeutic paradigm has the potential to completely transform the accepted standard of therapy for FLDs, providing reassurance and confidence in its effectiveness.
